# Narrowing the Differential: A Unique Case of Dystrophic Epidermolysis Bullosa

**DOI:** 10.1155/crpe/5515564

**Published:** 2025-09-18

**Authors:** Lauren Yacobucci, Carli Edwards, Annika Van Oosbree, Roger Newman

**Affiliations:** Division of Maternal Fetal Medicine, Medical University of South Carolina, Charleston, South Carolina, USA

## Abstract

Dystrophic epidermolysis bullosa (DEB) is a rare inherited skin disorder characterized by mechanical stress-induced blistering and skin erosion. Diagnosis is confirmed through molecular genetic testing, typically identifying mutations in the *COL7A1* gene. DEB can mimic other neonatal dermatologic conditions, making early identification challenging. We report a case of a male infant delivered at 35 weeks and 6 days via cesarean delivery to a mother with a complicated medical and obstetric history, including sickle cell disease and intrauterine fetal demise of one twin. At birth, the infant exhibited denuded skin on the right lower extremity and later developed erosions at peripheral IV sites. Initial differential diagnoses included infectious etiologies and Type V aplasia cutis. Infectious workup was unremarkable. An epidermolysis bullosa genetic panel identified a heterozygous pathogenic variant in *COL7A1* (c.6007G > A, p.Gly2003Arg), confirming the diagnosis of dominant DEB. The infant was managed with supportive wound care and discharged in stable condition with dermatology and genetics follow-up. This case underscores the importance of considering DEB in the differential diagnosis of neonatal skin lesions, especially in the context of a complex perinatal history. Early recognition and genetic confirmation are essential for appropriate management and family counseling.

## 1. Introduction

Epidermolysis bullosa (EB) is a rare, inherited genetic disorder characterized by blistering of the skin and mucous membranes precipitated by mechanical stress [1]. EB is classified into four major subtypes based on the level of tissue separation, namely, EB simplex (EBS), junctional EB (JEB), dystrophic EB (DEB), and Kindler syndrome. DEB is extremely rare, with a prevalence of approximately 19 per one million live births [2]. The prevalence also appears to be similar in males and females. Several conditions can present similarly to EB, so a high clinical suspicion is necessary when evaluating individuals with dermatological lesions. The definitive diagnosis of EB is made by molecular genetic testing for specific gene mutations [1]. Here, we present a case of a mother whose infant was born with unexpected desquamating skin lesions who was found to have DEB, and the clinical history leading to this diagnosis.

## 2. Case Presentation

The mother in this case is a 27-year-old G10P0181 with a spontaneous dichorionic-diamniotic twin gestation. She had a complicated past medical history which included recurrent early pregnancy loss with a negative antiphospholipid antibody syndrome workup. She also had a spontaneous twin gestation in her prior pregnancy, with an intrauterine fetal demise of one twin, followed by development of severe preeclampsia requiring cesarean delivery at 26 weeks gestation. Her pregnancy was also complicated by maternal sickle cell disease with resultant sickle cell retinopathy and avascular necrosis of the femoral head. Throughout her pregnancy, she required frequent admissions for pain control, titration of her medication regimen, and intermittent blood transfusions. Her medication regimen consisted of extended-release morphine sulfate (135 mg/day) and immediate release morphine (15 mg) as needed for breakthrough pain. She also received low dose aspirin for preeclampsia prophylaxis, as well as folic acid and oral iron supplementation. Lastly, given her elevated risk of venous thromboembolism, she was prescribed prophylactic low-molecular-weight heparin (40 mg/0.4 mL daily) through both in the antepartum and postpartum periods.

Similar to her prior pregnancy, she was diagnosed with an intrauterine fetal demise of twin B at 21 weeks gestation. At that time, she had cell -free DNA and maternal serum alpha fetoprotein (AFP) testing. Her cell-free DNA results were indicative of a pregnancy at low risk for trisomy 13, 18, 21, or other sex chromosome aneuploidies. Maternal serum AFP was elevated at 6.54 MoM; however, this abnormal result was attributed to the twin demise, rather than a fetal anomaly. At 29 weeks gestation, twin A was diagnosed with severe fetal growth restriction. Doppler studies of the umbilical artery were followed closely, and they remained normal.

At 35 weeks and 6 days, she was admitted to the hospital with sustained severe range blood pressures requiring both intravenous and oral antihypertensive medications. Based on her elevated blood pressures, as well as a significantly elevated urine protein to creatinine ratio (10 mg/g), she met criteria for severe preeclampsia. Given her gestational age, the decision was made to proceed with delivery, and she underwent an uncomplicated repeat cesarean.

**Figure 1:**
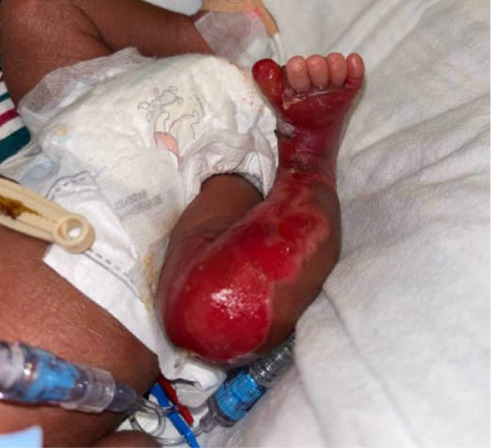
Lesions at time of delivery.

The male infant with a birthweight of 1990 g was noted to be limp and nonvigorous at birth, requiring intubation and surfactant delivery for poor respiratory effort. Additionally, at delivery, the infant was noted to have denuding of the right lower extremity (RLE) around the dorsal and plantar aspect of the foot, extending onto the right shin (Figure 1). He was admitted to the neonatal intensive care unit (NICU) due to respiratory distress syndrome and prematurity. The infant was able to be extubated to CPAP by day of life (DOL) 2 and then quickly weaned to room air. He remained stable on room air for the remainder of his admission.

**Figure 2:**
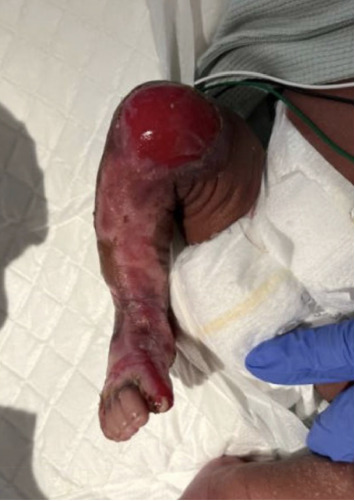
Healing lesions.

The initial differential diagnoses for the RLE skin lesion included Type V aplasia cutis, EB, and infectious etiologies such as staphylococcal scalded skin syndrome or neonatal herpes. The infant completed a 48-h sepsis evaluation which included prophylactic nafcillin and gentamicin, followed by a 5-day oral cefalexin course. Bacterial blood and wound cultures, herpes simplex PCR, and varicella zoster PCR were ultimately reassuring against an infectious etiology. The presumed diagnosis was Type V aplasia cutis given the unremarkable workup and twin gestation with a cotwin demise. On DOL 6, the infant was noted to have new erosion at the site of a peripheral intravenous catheter (PIV) prompting further investigation into EB and EB genetic panel was obtained. Wound care and dermatology were involved during the admission and provided wound care recommendations for the infant. This included applying a thick layer of Vaseline to the denuded area, with Mepitel as the primary dressing and continuation of mupirocin. The infant was discharged home on DOL 14 with genetics and dermatology follow-up. RLE skin lesions noted to be healing at time of discharge (Figure 2).

Soon thereafter, the EB panel resulted positive for a heterozygous pathogenic variant in the COL7A1 gene (c.6007G > A, p. Gly2003Arg). This variant has been reported in several individuals with autosomal dominant or autosomal recessive EB dystrophica. The particular variant identified in this infant's genetic panel, in addition to the clinical presentation, is consistent with dominant DEB (DDEB) (given that the infant is a heterozygote) if found to be an inherited mutation. Parental testing is unfortunately not available to confirm whether this represents a spontaneous or inherited mutation.

Written informed consent was obtained from the patient for publication of this case report and accompanying images.

## 3. Discussion

DEB is a rare genetic skin disorder characterized by blistering of the skin and mucous membranes which usually present at birth. The two major categories of DEB are based on the inheritance pattern, namely, recessive DEB (RDEB), or DDEB. Each category is further divided into multiple subtypes depending on the extent of type VII collagen expression and clinical manifestations of the disease [1, 3].

The worst presentation is generalized, severe RDEB which was previously known as Hallopeau–Siemens. RDEB has a significantly reduced amount of Type VII collagen leading to a number of extracutaneous manifestations including dysfunctions of the renal, cardiac, gastrointestinal, and genitourinary systems [1, 3]. As there is mucus membrane involvement with RDEB, these patients may also have manifestations within the esophagus, eyes, and anal canal. DEB overall, however, exists on a spectrum ranging from severe manifestations such as this, to relatively transient manifestations in the neonatal period, known as DDEB or RDEB bullous dermolysis of the newborn [1, 3].

Clinical suspicion must remain high in order to diagnose DEB given the broad differential diagnosis for skin conditions in infancy, as well as the fact that absence of a known family history of DEB does not preclude its diagnosis [3]. Infectious etiologies such as herpes simplex, syphilis, and staphylococcal skin manifestations including staphylococcal scalded skin syndrome and staphylococcal pyoderma must be ruled out. Several other skin disorders may present at birth with their own unique constellation of symptoms including ectodermal dysplasia, epidermolytic ichthyosis, and incontinentia pigmenti. In this unique case, given the history of a twin gestation with an IUFD of one twin, Type V aplasia cutis must also be ruled out. This can typically be differentiated from EB based on its lack of mucosal involvement and by ordering the appropriate genetic testing, as was done in this case [4].

The diagnosis of DEB can be confirmed by combining the clinical manifestations along with molecular genetic testing. Genetic testing can confirm the presence of biallelic pathogenic variants or a heterozygous pathogenic variant in the *COL7A1* gene, the only gene in which pathogenic variants are known to cause DEB [3]. Over 400 mutations have been documented in this gene. Alternative options for diagnostic testing include skin biopsy with direct immunofluorescence for specific cutaneous markers with or without electron microscopy, as shown in [5].

Genetic diagnosis in our case revealed this infant was a positive heterozygote for a pathogenic sequence variant in COL7A1 defined as c.6007G > A. This genetic alteration is predicted to result in the amino acid substitution p.Gly2003Arg. This variant has been reported in several individuals with autosomal dominant or autosomal recessive EB dystrophica [6, 7]. The amino acid p.Gly2003Arg resides in the exon 73 and is within the triple helical domain of the COL7A1 protein (amino acids 1254–2783). Glycine substitution variants in the triple helical domain (Gly X-Y; especially in exons 73, 74, and 75) are predominant in autosomal DDEB; thus, this variant is interpreted as pathogenic [3].

In our case, blistering was limited to the RLE and wrist. Dystrophic nails, especially toenails, are common and may be the only manifestation of DDEB. Long-term effects can be concerning for any affected persons, even those with more benign variants. Squamous cell carcinoma (SCC) can develop in any EB patient and will typically present at an earlier age than in non-EB patients [1, 3, 5]. SCC is far more prevalent in RDEB than DDEB, with an incidence of about 6% in DDEB patients [8, 9]. It is therefore imperative to achieve early re-epithelialization of any wound and to prevent recurrence whenever possible. From a wound healing standpoint, blisters are recommended to be punctured to avoid wound desquamation, with skin being left in place to act as a natural barrier. Skin should be lubricated with Vaseline or bland ointments, and then, lesions are covered with nonstick dressings. Compressive and adhesive dressings must not be used as they can produce new blisters. Due to the increased risk of bacterial resistance and colonization, topical antibiotic ointments and antimicrobial dressings should be reserved for colonized wounds that fail to heal [1, 3, 5]. The most common colonizing organisms are S*taphylococcus aureus, Streptococcus pyogenes*, and *Pseudomonas aeruginosa.* [10].

No definitive treatments have been established for DEB, and symptomatic therapies and wound care are the mainstay of clinical management. Infants with EB face many barriers to adequate nutrition including the need for extra calories for wound healing, mucosal fragility leading to oral blisters and erosion, anemia from chronic blistering, and gastrointestinal reflux disease (GERD) [10]. Respiratory compromise can occur from GERD-induced acid damage to the epiglottis and posterior pharynx. Orogastric and nasogastric tubes are not recommended as these can cause pharyngeal erosions and esophageal trauma leading to strictures [10]. Thus, alternative routes for nutrition such as central catheters with administration of total parenteral nutrition may be required for these infants to obtain adequate caloric intake.

There are several new potential treatment options on the horizon which may change clinical management for DEB. Firstly, allogeneic bone marrow transplantation has shown promise in its capacity to migrate to the skin and differentiate into skin cells which produce Type VII collagen. Additionally, intradermal injection of normal human fibroblasts or gene-corrected RDEB fibroblasts has been found to restore the synthesis and stable deposition of Type VII collagen at the dermal–epidermal junction, by elevation of heparin-binding-EGF-like growth factor (HB-EGF) [1]. Another attractive approach is recombinant protein therapy. Since EB is a monogenic disease, corrective gene therapy remains the ideal option for definitive future treatment, with minimal adverse effects [1].

This case describes the diagnosis and management of DEB, a rare desquamative neonatal skin disorder. It reminds the clinician about the potential genetic diagnosis of DEB among the multiple other possibilities in a lengthy differential. In this specific case, that differential is further confused by the concurrent fetal demise of a dichorionic cotwin raising the possibility of Type V aplasia cutis; another rare disorder initially suspected in this case.

## Data Availability

The data that support the findings of this study are available from the corresponding author upon reasonable request.
